# Facile exfoliation of natural talc into separated mesoporous magnesium silicate nano-sheets for effective sequestration of phosphate and nitrate ions: characterization and advanced modeling

**DOI:** 10.3389/fchem.2025.1571723

**Published:** 2025-04-15

**Authors:** Walaa Gaber, Nabila Shehata, Ahmed M. El-Sherbeeny, Wail Al Zoubi, Ahmed Mehaney, Mostafa R. Abukhadra

**Affiliations:** ^1^ Environmental Science and Industrial Development Department, Faculty of Postgraduate Studies for Advanced Sciences, Beni-Suef University, Beni-Suef, Egypt; ^2^ Renewable Energy Science and Engineering Department, Faculty of Postgraduate Studies for Advanced Sciences, Beni-Suef University, Beni-Suef, Egypt; ^3^ Industrial Engineering Department, College of Engineering, King Saud University, Riyadh, Saudi Arabia; ^4^ Materials Electrochemistry Laboratory, School of Materials Science and Engineering, Yeungnam University, Gyeongsan, Republic of Korea; ^5^ Physics Department, Faculty of Science, Beni-Suef University, Beni-Suef, Egypt; ^6^ Applied Science Research Center, Applied Science Private University, Amman, Jordan; ^7^ Materials Technologies and their applications Lab, Faculty of Science, Beni-Suef University, Beni Suef, Egypt

**Keywords:** talc, exfoliation, silicate nano-sheets, adsorption, energetic, steric

## Abstract

Magnesium silicate nano-sheets were synthesized from natural talc by facile exfoliation and delamination methods as exfoliated product (EXTC) of 29.5 nm average pore diamter, enhanced surface area (103 m^2^/g), and adsorption perforamnces. The sucessful development of EXTC particles was followed based on different techniques and applied in effective sequestration of PO_4_^3-^ and NO_3_^−^ ions from water. The EXTC product as adsorbent demonstrates remarkable effectiveness for both PO_4_^3-^ (257.9 mg/g) and NO_3_^−^ (164.2 mg/g) as compared to several studied structures. Depending on the steric analysis of Monolayer equilibrium model, the interface of EXTC highly saturated with interactive receptors for the both ions but with higher abundant for PO_4_^3-^ (151.5 mg/g) as compared to NO_3_^−^ (61.5 mg/g). This resulted in higher aggregation effect during the uptake of NO_3_^−^ (4 ions per site) than PO_4_^3-^ (3 ions per site) which also donate the vertical orientation of these adsorbed ions and operation of multi-ionic sequestration mechanisms. The structure is highly recyclable and of significant safety and cane be applied in its spent or exhausted state as fertilizer. The energetic evaluation considering the Gaussian energy (<8.5 kJ/mol) as well as the sequestration energy (<4 kJ/mol), suggested the predominant impact of physical mechanisms (hydrogen bonds and electrostatic attraction), in addition to the impact of the weak chemical complexation. Furthermore, the thermodynamic functions declare the retention of these ions into the framework of EXTC by exothermic and spontaneous reactions.

## 1 Introduction

The contamination of freshwater resources poses a significant challenge to modern society, threatening both environmental sustainability and human health (Yang et al., 2022; Zourou et al., 2022). The World Health Organization (WHO) has issued an urgent warning that by 2025, over 50% of the global population is likely to experience severe water shortages (Zourou et al., 2022; Arab et al., 2022). Industrial, agricultural, and mining activities are the primary contributors to water pollution, releasing a wide range of pollutants, including bacteria, pesticides, heavy metals, fertilizers, pharmaceuticals, and dyes (Ighnih et al., 2024; Kusuma et al., 2024). Among these contaminants, phosphate (PO_4_^3-^) and nitrate (NO_3_^−^) ions are particularly problematic due to their detrimental effects on water quality. Even at relatively low concentrations (0.05 mg/L), phosphate ions in enclosed water bodies accelerate eutrophication, resulting in excessive algal growth and oxygen depletion (Shi et al., 2019a; Che et al., 2024). Similarly, nitrate contamination exceeding 50 mg/L can lead to severe health issues, including methemoglobinemia, nitrosamine formation, and blue baby syndrome, with the WHO-recommended limit set at 10 mg/L for drinking water (Wang et al., 2021; Abali et al., 2021; Onyango et al., 2010).

The urgent need for effective water treatment technologies has led researchers to explore adsorption as an efficient, cost-effective, and sustainable method for pollutant removal (Naat et al., 2021; Albukhari et al., 2021). Various adsorbent materials, such as biochar, zeolites, layered double hydroxides (LDHs), and metal oxides including MgO, Fe_2_O_3_, CeO_2_, and Ce/Zr bimetallic oxides, have been extensively studied for their potential in water purification (Khoshkalam et al., 2023; Liang et al., 2023; Ederer et al., 2023). The selection of an optimal adsorbent depends on several critical parameters, including production costs, synthesis techniques, precursor availability, adsorption efficiency, recyclability, kinetics, selectivity, and chemical stability (Fan et al., 2024; Abukhadra et al., 2020). Among the naturally occurring adsorbents, clay minerals and other phyllosilicates are widely preferred due to their abundance, low cost, and eco-friendly nature (Yang et al., 2022; Ifa et al., 2022; Sun et al., 2024). Functionalized clay-based nanomaterials have demonstrated exceptional efficiency in removing organic and inorganic pollutants owing to their high ion-exchange capacity, chemical stability, and reactive surface properties (Chen et al., 2021; Alqahtani et al., 2023a; Abdel Salam et al., 2022; Ahmed et al., 2024).

Clay minerals generally consist of layered silicate structures, which can be categorized into aluminum-rich and magnesium-rich compositions. While extensive research has focused on aluminum-based clays such as kaolinite and montmorillonite, the potential of magnesium silicate-based minerals, particularly talc, remains underexplored (Ighnih et al., 2023a; Dardir et al., 2018; Meng et al., 2020). To enhance the adsorption properties of clay minerals, several modification techniques have been applied, including acid or alkaline activation, thermal treatment, incorporation of metal oxides, exfoliation, and organic functionalization (Meng et al., 2020; Ighnih et al., 2023b). Among these methods, exfoliation has emerged as a particularly effective strategy for increasing the surface area, porosity, and reactivity of clay minerals, thereby significantly enhancing their adsorption capacity (Sabry and AbdulAzeez, 2014; Singh and Dutta, 2019; Alqahtani et al., 2023b).

Exfoliation involves the delamination of layered minerals into nanoscale sheets, producing high-surface-area materials with improved adsorption and dispersion properties. This process significantly increases the availability of reactive sites, enhances ion-exchange capabilities, and facilitates chemical interactions with target contaminants (Alqahtani et al., 2023b; Naciri et al., 2024; Tan et al., 2018; Abukhadra et al., 2021). Chemical exfoliation, often assisted by ultrasonication, has been widely applied to aluminum-rich clays such as montmorillonite and kaolinite to produce high-performance nanostructured adsorbents (Zhu et al., 2022; Das et al., 2023). However, despite the advantages of exfoliation, limited studies have explored the exfoliation of magnesium-rich clay minerals, particularly talc.

Talc (Mg_3_(Si_4_O_10_)(OH)_2_) is a naturally occurring magnesium phyllosilicate with a TOT (tetrahedral-octahedral-tetrahedral) layered structure, where silica tetrahedral layers sandwich a brucite (Mg(OH)_2_) octahedral layer (Zhang et al., 2024). This structure contributes to talc’s high chemical stability, low cost, and biocompatibility (Zhan et al., 2024; Wu et al., 2023; Burkett et al., 2022; Allah et al., 2023). Talc has been investigated as an adsorbent for pollutants such as methylene blue dye (Bilgiç et al., 2022), Cr(VI) (Nayl et al., 2022), Pb(II) (Abass et al., 2022), and rare earth elements (Zhang et al., 2024). However, conventional talc materials exhibit limitations in adsorption performance due to their relatively low surface area and poor dispersibility in aqueous media. The exfoliation of talc into distinct magnesium silicate nanosheets has the potential to overcome these limitations by significantly increasing the number of accessible reactive sites and enhancing adsorption efficiency.

Despite its potential, talc exfoliation remains an underexplored area of research, particularly for its application in water purification. Previous studies involving talc exfoliation have primarily focused on polymer intercalation or liquid-phase exfoliation without structural or morphological validation of the resulting nanosheets as well as the investigation of its adsorption performances. The exfoliation of talc into magnesium silicate nanosheets offers multiple advantages. The increased surface area and exposed functional groups of the exfoliated layers significantly improve adsorption capacity for various pollutants. Exfoliated nanosheets also exhibit better aqueous stability, allowing for more effective interactions with contaminants in solution. The increased number of active sites facilitates stronger binding interactions with dissolved pollutants.

This study aims to introduce an innovative, facile exfoliation method to transform talc into distinct magnesium silicate nanosheets with enhanced physicochemical properties. Unlike previous studies that lacked structural confirmation of exfoliated talc, this research provides a comprehensive characterization of the resulting nanosheets and systematically evaluates their adsorption performance. The synthesized nanosheets were systematically evaluated for their phosphate (PO_4_^3-^) and nitrate (NO_3_^−^) adsorption performance under various experimental conditions. For the first time, the adsorption properties of exfoliated talc-based magnesium silicate nanosheets were analyzed using both classical adsorption isotherms and advanced statistical physics-based modeling. The study comprehensively examined the adsorption mechanisms at the adsorbent/adsorbate interface, integrating key parameters such as active site density, saturation uptake capacity, single-site occupancy, adsorption energy, and thermodynamic functions (entropy, enthalpy, internal energy, and adsorption energy). This approach provided a deeper understanding of the adsorption behavior of exfoliated talc and its potential for application in water treatment technologies. This research aims to establish exfoliated talc-based magnesium silicate nanosheets as an advanced, high-performance, and cost-effective adsorbent for water purification. The findings contribute to expanding the application of magnesium-rich silicates as viable alternatives to conventional aluminum-based clay adsorbents, promoting the development of sustainable water treatment materials with enhanced adsorption efficiencies.

## 2 Experimental work

### 2.1 Materials

The starting talc mineral was supplemented by El-Nasr Mining Company as mining raw minerals from Bir Meseh area, South of Shalatin, Eastern Desert, Egypt. Chemically, the investigated talc sample composed of 46.35% SiO_2_, 25.53% MgO, 9.54% CaO, 3.86% Al_2_O_3_, 2.16% Fe_2_O_3_, 0.13% P_2_O_5_, 0.02% K_2_O, 0.28% TiO_2_, 0.12% SO_3_, 0.18% Na_2_O, and 11.83% loss of ignition (L.O.I.). Throughout the talc transformation processes, dimethyl sulfoxide (DMSO) (>99.5%), NaOH pellets (97%), methanol (>99.9%), and cetyltrimethylammonium bromide (CTAB) (>98%) had been obtained via Sigma-Aldrich and Egypt. Standard solutions of phosphate and nitrate had been provided by Sigma-Aldrich Company with 1,000 mg/L concentrations.

### 2.2 Exfoliation of talc

The talc-layered units were exfoliated using a simple chemical expansion process as outlined by Allah et al. (2023). Talc was ground and pulverized using a ball mill over a period of 8 h to yield a fine powder having a particle size ranging from 20 to 100 µm. Following that, a conventional magnetic stirring device was implemented to meticulously mix the pulverized talc (10 g) together with DMSO solution with a maintained volume of 50 mL, and this continued up to 6 h. The method previously mentioned is essential for breaking the existing hydrogen bonds that bind the various stacked silicate layered units comprising the talc structure. After treating the stage of talc with DMSO, its modified particles were washed by methanol for about 15 min. This procedure was performed repeatedly for five cycles to entirely eradicate the existing DMSO molecules and replace them effectively with the other forms of integrated methanol molecules, producing methanol intercalated talc or methoxy talc. The methoxy talc particles were dispersed inside a CTAB-based aqueous solution (20 g in 50 mL of distilled water). The mixture was extensively mixed and homogenized via a combined blending system comprising a magnetic stirring device (1,000 rpm) alongside a sonication generator (240 W) over 48 h. This step aimed mainly to separate and exfoliate the magnesium silicate-layered units of talc into individual nanosheets (EXTC). The generated EXTC nanoparticles were then carefully rinsed using distilled water, delicately dried at 70°C over 15 h, and kept for complete characterization and applications.

### 2.3 Characterization instruments

The crystal phases and crystalline characteristics were evaluated and investigated by employing X-ray diffraction (XRD) patterns obtained via the PANalytical-Empyrean X-ray diffractometer. The determination limits of the 2 Theta angles using the diffractometer extend from 0° to 80°. The alteration in the fundamental chemical groups throughout the fabrication stages was assessed employing a Shimadzu FTIR-8400S spectrometer, which encompasses the measurement range from 400 to 4,000 cm^−1^. The surface features of the synthesized zeolite as well as raw talc were examined by employing a Gemini Zeiss Ultra 55 scanning electron microscope. Prior to imaging, the exteriors of the materials under investigation were prepared by coating them with gold sheets by spraying. The porosity extents and specific surface area were measured using a surface area analyzer (BELSORP-miniX; S/N: 10,039; Software version: Version 1.1.3.1) after evacuating any gases from the sample. The incorporated sample mass, manifold volume, free space are 0.2106 g, 12.376 cm^3^, and 18.004 cm^3^, respectively. The analysis has been completed, implementing the standard N_2_ adsorption and desorption isotherm which was measured at 77 K. The specific surface area was determined based on the Brunauer-Emmett-Teller (BET) method (P/P0 = 0.020806 to 0.4747 and p/Va (p_0_-p) = 0.1653–2.1546). Pore size distribution was obtained by the Barrett-Joyner-Halenda (BJH) method.

### 2.4 Batch adsorption experiments

The adsorption experiments of PO_4_^3-^ and NO_3_^−^ by synthesized EXTC have been performed in batch mode, implementing the effects of pH (2–10), pollutants content (50–400 mg/L), and retention time (30–420 min). The experiments had been performed in three separate experiments, utilizing a constant volume of 100 mL and EXTC dose of 0.2 g/L. The adsorption equilibrium experiments were evaluated at various temperature settings, specifically 303 K, 313 K, and 323 K. Following the completion of each examination’s equilibrium period, the treated solutions were filtered to exclude the solid particles of EXTC and determine the remaining contents of PO_4_^3-^ and NO_3_^−^. The residual contents of PO_4_^3-^ and NO_3_^−^ were measured by applying Dionex DX-120 ion chromatography device. The results were subsequently employed to calculate the adsorption capacities of EXTC for the two pollutants, following Equation 1. The PO_4_^3-^ and NO_3_^−^ standards that were utilized during the measurement procedures were purchased from Merck Company (Germany) and then certified by the National Standard and Technology Institute (NIST). The characters Q_e_, C_o_, C_e_, V, and m in the formula represent the adsorption capacity (measured in mg/g), the starting levels (measured in mg/L) of the studied ions, the residual concentrations (measured in mg/L) of PO_4_^3-^ and NO_3_^−^, the volume (in mL) of the aqueous solutions contaminated with the target ions that were tested, and the dose (measured in mg) of EXTC.

$$Q_{e\;\left(mg/g\right)}=\frac{\left(C_{o}-C_{e}\right)V}{m}$$ (1)

### 2.5 Conventional and modern equilibrium investigations

The adsorption of PO_4_^3-^ and NO_3_^−^ using EXTC has been described using well-established traditional kinetics, classic equilibrium, and updated isotherm investigations in accordance with the theoretical statistical physics hypothesis (Supplementary Table S1). The kinetic and conventional isotherm modeling have been assessed employing the non-linear fitting levels of the retention data of PO_4_^3-^ and NO_3_^−^. The evaluation implemented the parameters of the coefficient (*R* ^2^) (Equation 2) and Chi-squared (χ^2^) (Equation 3). The nonlinear fitting qualities with the modern isotherm models’ descriptive equations and the remediation results of PO_4_^3-^ and NO_3_^−^ have been examined using the determination coefficient (*R* ^2^) and root mean square error (RMSE) (Equation 4). The variables m′, p, Qi_cal_, and Qi_exp_ in the equation correspond to the outcomes of PO_4_^3-^ and NO_3_^−^ sequestration, parameters affecting the sequestration reaction, predicted capacities of ions sequestration, and determined capacities of ions sequestration, respectively.

$$R^{2}=1-\frac{\sum {\left(Q_{e,\exp}-Q_{e,cal}\right)}^{2}}{\sum {\left(Q_{e,\exp}-Q_{e,mean}\right)}^{2}}$$ (2)

$$\chi^{2}=\sum \frac{{\left(Q_{e,\exp}-Q_{e,cal}\right)}^{2}}{Q_{e,\text{cal}}}$$ (3)

$$\text{RMSE}=\sqrt{\frac{\sum_{i=1}^{m}{\left({\text{Qi}}_{\text{cal}}-{\text{Qi}}_{\exp}\right)}^{2}}{m^{\prime }-p}}$$ (4)

## 3 Results and discussion

### 3.1 Characterization results

#### 3.1.1 XRD analysis

**FIGURE 1:**
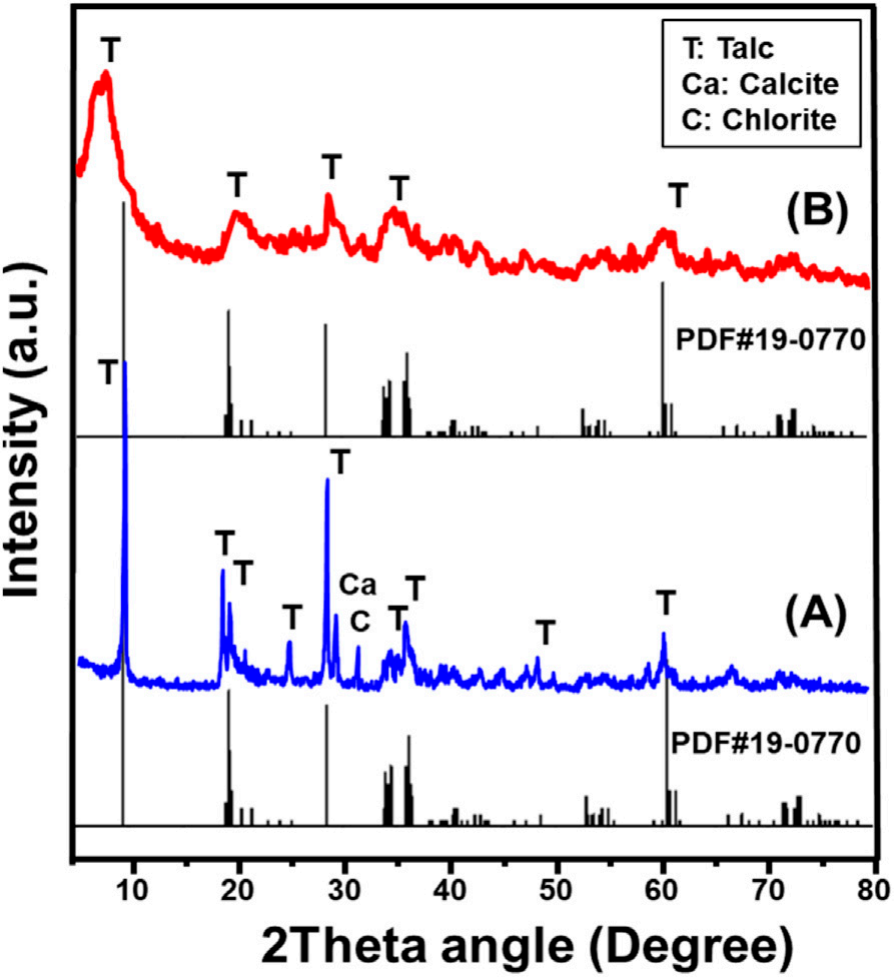
XRD patterns of raw talc **(A)** and exfoliated talc sheets (EXTC) **(B)**.

The X-ray diffraction (XRD) analysis provided strong evidence of the successful transformation of the layered silicate structure of talc into a tecto-silicate framework, corresponding to the formation of synthesized zeolite phases (Figure 1). The XRD pattern of the initial talc precursor clearly indicated its highly crystalline nature, characterized by well-defined peaks attributed to talc (PDF#29–1493), along with minor mineral impurities, including chlorite and calcite (PDF#00-005-0586) (Figure 1A). The distinct diffraction peaks observed at 2θ angles of 9.48° (002), 19.21° (004), 28.62° (006), 36.31° (132), and 60.50° (−331) confirmed the presence of a monoclinic crystal system with a d-spacing value of 9.32 Å, consistent with the chemical composition of Mg_3_Si_4_O_10_(OH)_2_ (PDF#19-0770; PDF#29-1,493). These results validate the well-ordered silicate arrangement in the raw talc structure, which plays a critical role in its physicochemical stability.

Following the exfoliation process, the XRD pattern of the synthesized exfoliated nanomagnesium silicate sheets (EXTC) exhibited significant structural alterations, indicating the substantial impact of the applied modification technique. A notable decrease in peak intensity, along with the disappearance of several dominant diffraction peaks of talc, was observed, suggesting extensive crystalline disruption and delamination effects. The remnant peaks of talc appeared in significantly reduced intensities and were shifted, confirming partial amorphization and exfoliation of the silicate layers. One of the most prominent changes was the transformation of the main diffraction peak at 9.48° (002), which became significantly broadened and shifted to a lower angle of 7.63° (Figure 1B). This pronounced shift strongly indicates the breakdown of the ordered silicate framework, leading to increased interlayer spacing and successful exfoliation of the talc sheets.

The observed XRD modifications confirm that the exfoliation process effectively separated the stacked silicate layers, converting talc into highly delaminated nanosheets with significantly altered crystallinity. These structural changes directly influence the physicochemical properties of the exfoliated material, potentially enhancing its reactivity, surface area, and adsorption capabilities. The reduction in crystallinity, coupled with the increased disorder of the silicate layers, suggests that the exfoliated product (EXTC) exhibits improved accessibility to active sites.

#### 3.1.2 FT-IR analysis

**FIGURE 2:**
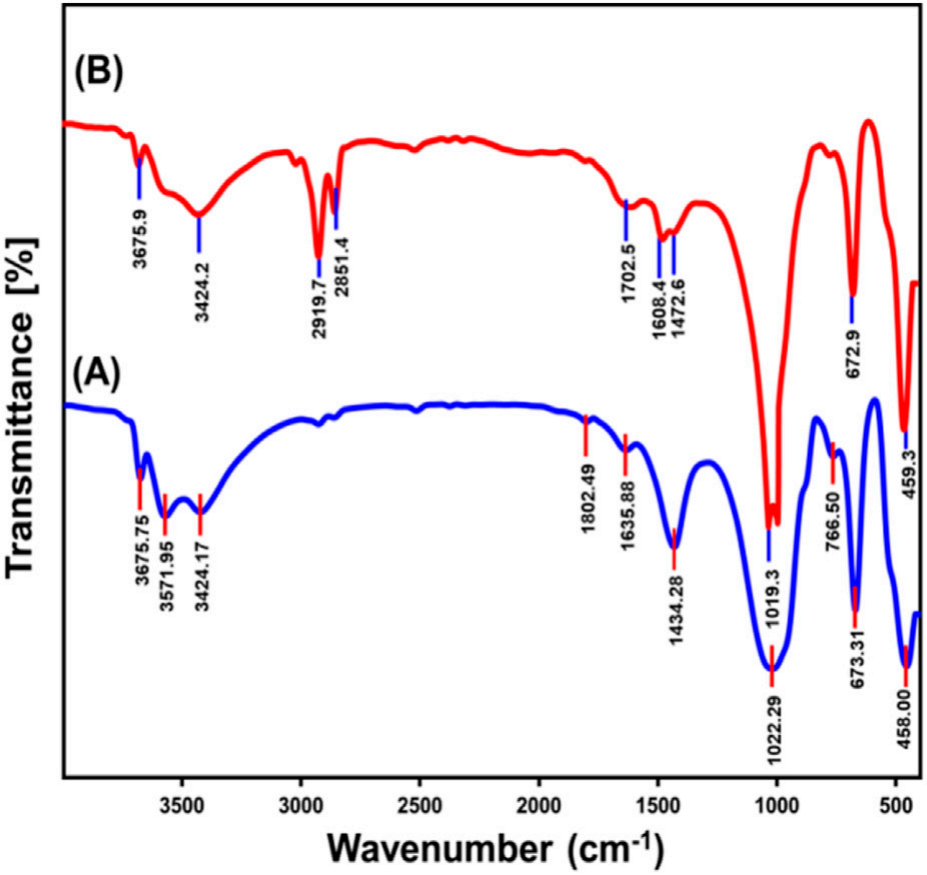
FT-IR spectra of raw talc **(A)** and exfoliated talc sheets (EXTC) **(B)**.

The FT-IR spectral analysis of raw talc and exfoliated nanomagnesium silicate sheets (EXTC) (Figure 2) revealed distinct modifications in the chemical composition and structural framework, providing key insights into the transformation process. The spectral profile of pristine talc exhibits characteristic absorption bands corresponding to fundamental functional groups. These include the stretching vibrations of Mg-OH groups along the edges of the octahedral units (3675.7 cm^−1^), weakly bonded hydroxyl groups (3571 cm^−1^), and interlayer stretching attributed to hydrogen bonding (3424 cm^−1^). The Si–O bond stretching at 1,022.3 cm^−1^, the bending vibration of adsorbed water molecules (1,635 cm^−1^), antisymmetric Si-O-Si stretching (766 cm^−1^), Si-O-Mg stretching (673 cm^−1^), and Si-O-Si bending vibrations (458 cm^−1^) further confirm the silicate framework of talc (Figure 2A) (Zhang et al., 2024; Yang et al., 2006; Hariram et al., 2019). Additionally, the observed band at 1,434 cm^-1^ is associated with the bending vibrations of CO_3_^2-^ groups, which likely result from partial carbonation of talc due to interactions with atmospheric CO_2_ (Zhang et al., 2023; Xie et al., 2024).

Following exfoliation, the FT-IR spectrum of EXTC exhibited several notable changes, reflecting significant chemical and structural transformations (Figure 2B). A marked shift and reduction in intensity were observed in the characteristic bands of talc, particularly in the Si-O stretching vibration (which shifted from 1,022 cm^−1^ to 1,019 cm^−1^) and the Si-O-Mg stretching band (which shifted slightly from 673 cm^−1^ to 672.9 cm^−1^). Furthermore, the Si-O-Si antisymmetric stretching vibration at 766 cm^−1^ exhibited substantial attenuation, indicating disruption within the silicate framework. These spectral shifts strongly suggest that exfoliation induced a structural reorganization, leading to the delamination of the silicate layers and partial breakdown of the crystalline order (Allah et al., 2023; Shawky et al., 2019). The splitting of the Si-O absorption band into two distinct regions around 1,000 cm^−1^ further supports this hypothesis, signifying the detachment and peeling of silicate sheets into individual nanoscale layers. This process disrupts both the silica tetrahedral and magnesium octahedral units, causing observable distortions in the crystal lattice. Moreover, the presence of distinct bands at 2,919 cm^−1^ and 2,851 cm^−1^ in the EXTC spectrum provides strong evidence of the incorporation of CTAB molecules during the exfoliation process (Figure 2B) (Allah et al., 2023). Additionally, these bands could be linked to secondary carbonation effects, wherein atmospheric CO_2_ interacts with the exfoliated material, resulting in surface-adsorbed carbonaceous species.

The observed spectral variations validate the successful exfoliation of talc into distinct magnesium silicate nanosheets, with significant structural reconfiguration and enhanced surface functionality. These findings indicate that the exfoliated EXTC material exhibits an improved reactivity profile and enhanced adsorption potential, making it a promising candidate for advanced applications in adsorption, catalysis, and environmental remediation.The Si–O stretching vibration at 1,019 cm^−1^ (shifted from 1,022 cm^−1^ in raw talc) and the Si–O–Si bending vibrations at 766 cm^−1^ and 458 cm^−1^ suggest structural deformation, leading to enhanced ion exchange capabilities. The weakening of these bands indicates increased exposure of silanol (-SiOH) groups, which can facilitate electrostatic interactions with negatively charged PO_4_^3-^ and NO_3_^−^ anions (Zhang et al., 2024; Yang et al., 2006; Hariram et al., 2019). The Si–O–Mg vibration at 672.9 cm^−1^ (shifted from 673 cm^−1^ in raw talc) is indicative of structural rearrangements that enhance the material’s affinity for dissolved ions. The increased availability of Mg^2+^ cations promotes electrostatic interactions and complexation with anionic species (Allah et al., 2023; Shawky et al., 2019). The strong broad band observed at 3424 cm^−1^ corresponds to hydrogen-bonded hydroxyl groups play a crucial role in forming hydrogen bonds with adsorbed anions. The detected shifts and intensity reductions in the hydroxyl stretching bands at 3675.7 cm^−1^ and 3571 cm^−1^ suggest modifications in the coordination environment of Mg–OH groups, increasing their reactivity toward ion adsorption. These functional groups collectively enhance the adsorption efficiency of EXTC, providing an advanced material with high selectivity and affinity for phosphate and nitrate removal.

#### 3.1.3 SEM and HRTEM analyses

**FIGURE 3:**
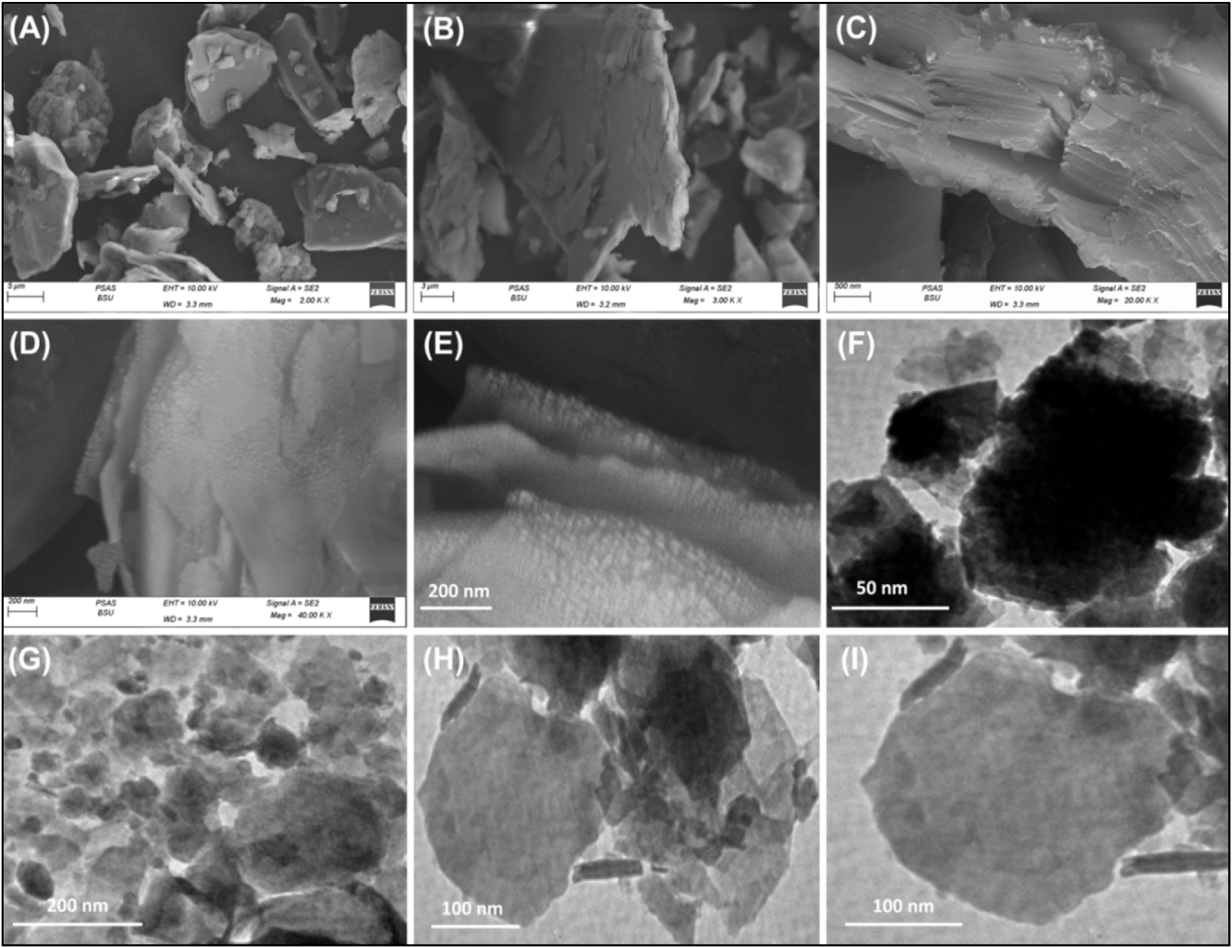
SEM images of raw talc **(A, B)**, high magnification image for the layered units of talc **(C)**, SEM images of exfoliated talc **(D, E)**, HRTEM image of raw talc **(F)**, and HRTEM images of the synthesized exfoliated talc particles **(G–I)**.

These structural and chemical transformations are accompanied by substantial alterations in the geometry and morphology of talc particulates, as demonstrated by scanning electron microscopy (SEM) and high-resolution transmission electron microscopy (HRTEM) analyses (Figure 3). The SEM images confirm the successful exfoliation of talc into distinct silicate layers, highlighting significant morphological changes. In its raw form, talc appears as flaky grains with well-defined plate-like structures and relatively smooth surfaces, which are characteristic of its natural crystalline morphology (Figures 3A,B). High-magnification images further reveal that the talc layers are tightly stacked, forming compact aggregates typical of phyllosilicate minerals (Figure 3C). Following exfoliation, the talc layers undergo noticeable delamination, with the individual sheets appearing split, separated, and displaced from each other, a characteristic phenomenon observed in phyllosilicate exfoliation (Figures 3D,E). This morphological transformation is indicative of the successful weakening of interlayer forces, leading to the formation of loosely arranged nanosheets. The HRTEM images provide further confirmation of these structural modifications. The raw talc particles exhibit a dense and compact arrangement, with dark gray to black contrasts in the TEM images, signifying the thickness of multiple overlapping layers (Figure 3F). After exfoliation, a striking transition is observed, as the talc particles transform into ultra-thin mono-sheets and isolated single layers, appearing as light green-toned structures in the TEM images (Figures 3G–I). Additionally, the presence of some rod-like structures suggests the initial formation of scroll-like morphologies, indicative of an advanced stage of morphological evolution during the delamination process. These findings confirm the extensive breakdown of the stacked silicate layers, leading to an increase in surface area, exposure of active adsorption sites, and enhanced dispersibility, which are critical factors for improving the material’s performance in adsorption.

#### 3.1.4 BET analysis

**FIGURE 4:**
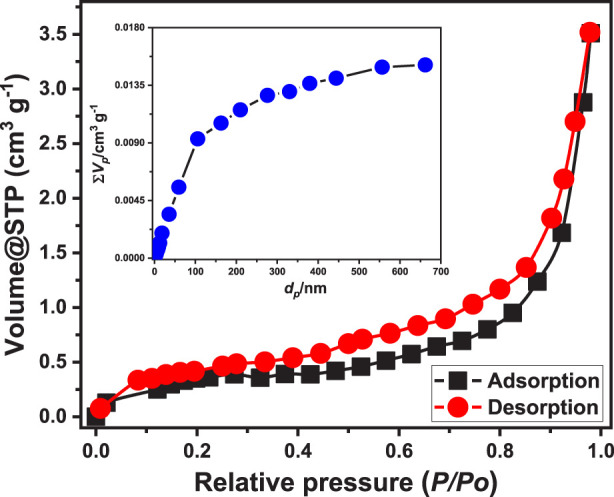
The nitrogen adsorption/desorption isotherm curve and pore size distribution properties of EXTC.

Texturally, the properties of synthesized EXTC particles were evaluated based on their nitrogen adsorptiopn/desorption isotherm curve (Figure 4). The curve can be classified as a type II isotherm according to the basics of the International Union of Pure and Applied Chemistry (IUPAC). Additionally, the curve exhibits a noticeable hysteresis loop of type H3 from P/P_0_ = 0.20 to 1 (Chen et al., 2018). Such criteria reflect the existence of the mesoporous structure of EXTC, which can be assigned to the non-rigid aggregation of plate-like particulates or slit-shaped pores (Chen et al., 2018). The size distribution curve reflected the porpus properties of the structure with average pore diameter is 29.5 nm and median pore diamter of 83.9 nm (Figure 4). Regarding the measured specific area, the synthetic EXTC possesses an execllent value equal to 103 m^2^/g. This qualifies the synthetic strucrure to be applied effectively, either as an adsorbent for water contaminants or as a heterogeneous catalyst, in addition to other applications as a drug delivery system or as catalyst support, considering its porosity and significant surface area.

### 3.2 Adsorption results

#### 3.2.1 Effect of pH

**FIGURE 5:**
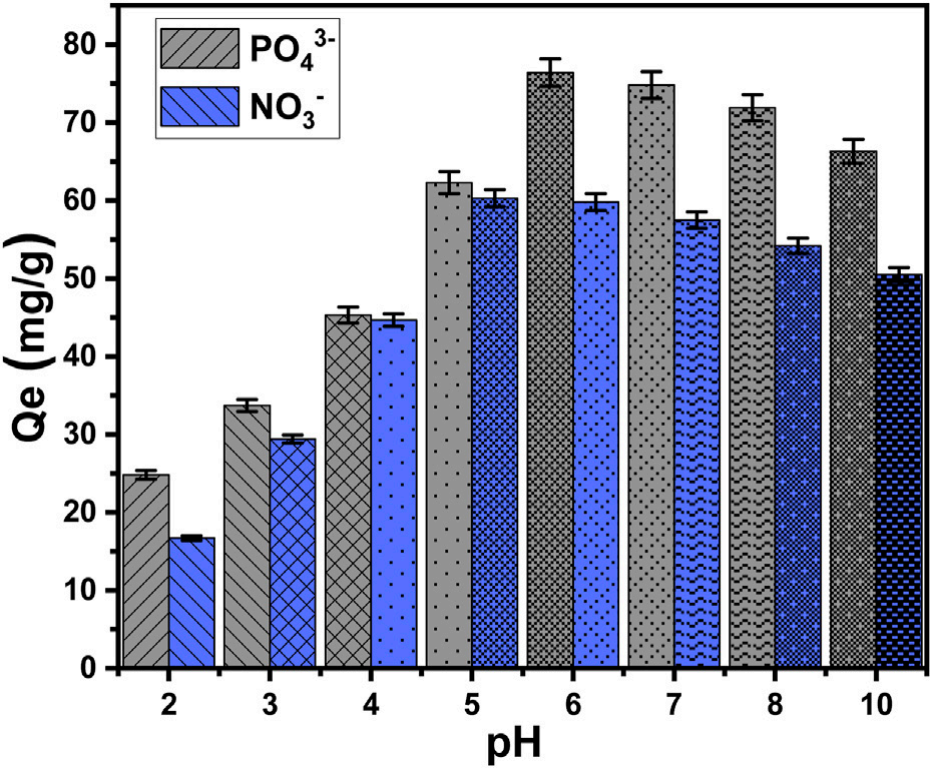
The impact of the pH levels on the uptake performances of PO_4_^3-^ and NO_3_^−^ by EXTC.

The pH of the aqueous solutions corresponding to the soluble chemicals exerts a considerable impact on both the exterior charges of EXTC and the ionizing or speciation behaviors of the tested chemical ions (Kobylinska et al., 2023). The pH impact was detected throughout a pH range of 2–10. The main effective testing variables were kept constant at specific values: an interaction period of 120 min, a temperature of approximately 303 K, a total volume of 100 mL, a concentration of pollutants at 100 mg/L, and a dosage of EXTC at 0.20 g/L. The adsorption results for PO_4_^3-^ and NO_3_^−^ show a significant increase whenever higher pH values are tested, reaching a pH of 6 and 5, respectively. At this specific pH setting, the ability of PO_4_^3-^ to be adsorbed is 76.4 mg/g (pH 6), whereas the ability of NO_3_^−^ to be adsorbed is 60.3 mg/g (pH 5) (Figure 5). As a result, the particulates of EXTC have sufficient capacity to be used as efficient adsorbents in real wastewater purification technologies, matching the pH range of 6–9, as reported by the United States Environmental Protection Agency (EPA) for the remediation of industrially polluted effluents (Jiang et al., 2020). The continuous increment in the pH levels higher than the mentioned values (pH 6 (PO_4_^3-^) and pH 5 (NO_3_^−^)) resulted in remarkable declination in the determined adsorption capacity of EXTC for the two chemical ions. The mentioned uptake behavior could potentially be attributed to the ionization as well as the speciation tendency of both PO_4_^3-^ and NO_3_^−^ ions and the dominant charges onto the surfaces of EXTC during the adsorption reactions at different pH situations.

In acidic conditions with a pH less than 3, phosphate (H_3_PO_4_) is neutral. At pH values ranging from 3 to 6, it was present in its acidic H_2_PO_4_^−^ form. Under conditions of neutrality and alkalinity, when the pH exceeds 6, phosphate is present in two more acidic forms, particularly HPO_4_^2-^ and PO_4_^3-^ (El-Sherbeeny et al., 2021a; Fu et al., 2018). EXTC has poor uptake efficacy in acidic settings due to the ionizing activities of phosphate and the protonation/deprotonation features of its chemical groups within the evaluated pH ranges. The reason for this behavior may be attributed to the hydrogenated external interface of EXTC, which displays a low electrostatic affinity for the neutral species of soluble phosphate (H_3_PO_4_). As the pH rose, the electrostatic attractive effects progressively intensified, correlating with the existence and development of the acidic forms as the dominant species (H_2_PO_4_^−^). This resulted in a significant increase in EXTC’s recognized sequestration capacity, up to a pH 6 threshold value. Moreover, the deprotonation of the EXTC at pH values ranging from neutral to alkaline resulted in the emergence of the structural chemical groups with net negative charges. Therefore, these groups have notable repulsive properties against mostly existing phosphate forms, particularly HPO_4_^2-^ and PO_4_^3-^ (Shi et al., 2019b). Within significantly acidic environments, the protonation of NO_3_^−^ leads to a notable decrease in the electrostatic force that exists between hydrogenated or protonated groups of EXTC and hydrogenated ions of nitrate (HNO_3_) (Li et al., 2020). The regular de-protonation of the resulting HNO_3_ with the systematic rise in the pH values corresponds to an increase in their electrostatic attractions to the still protonated chemical groups of EXTC until pH 5 (Li et al., 2020; Rahdar et al., 2021). After pH 5, the level of existing negative -OH ions on the exterior of the deprotonated surface of EXTC increases, exhibiting electrostatic repellent properties with NO_3_^−^ ions (Li et al., 2020; He et al., 2020).

#### 3.2.2 Retention interval

**FIGURE 6:**
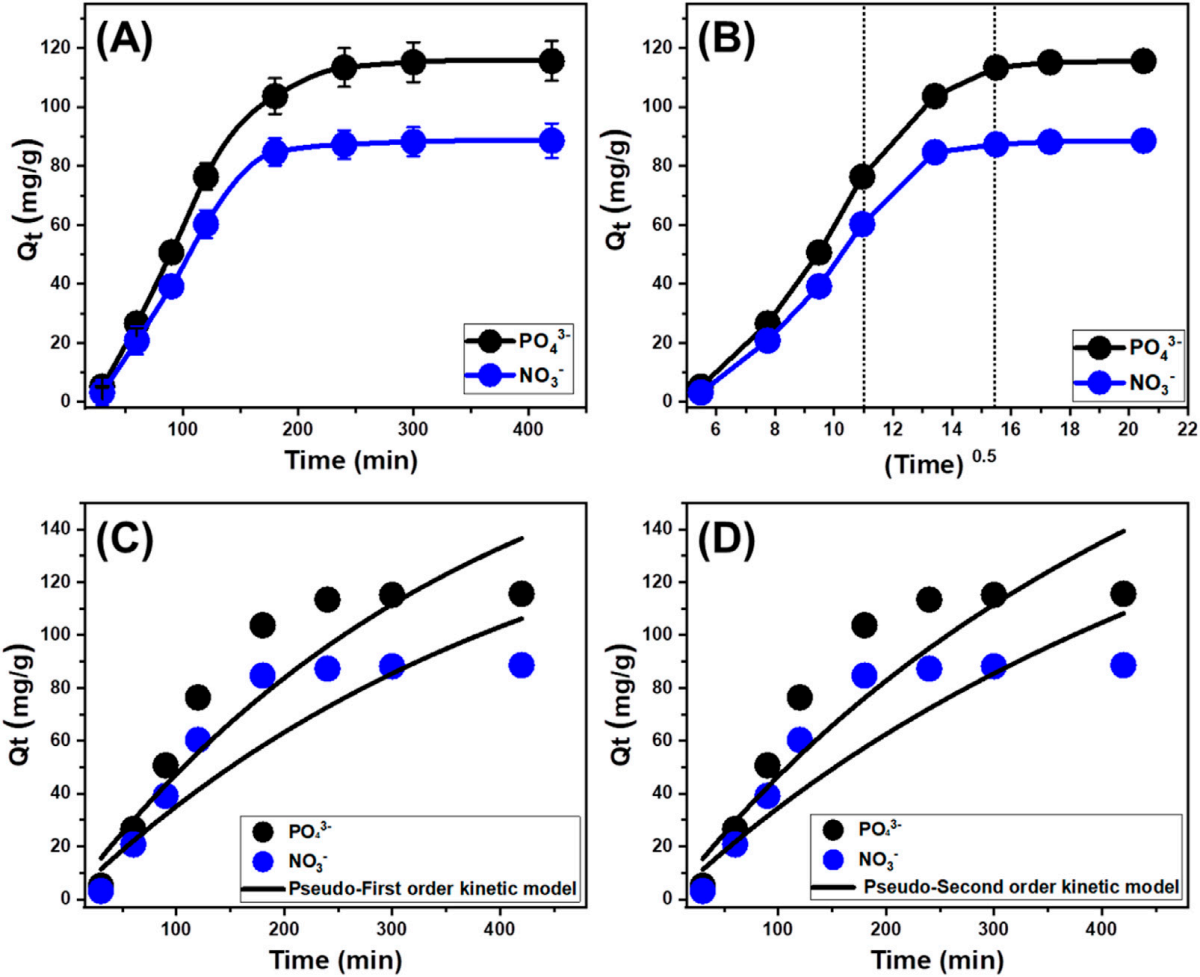
The experimental impact of contact duration of the uptake of PO_4_^3-^ and NO_3_^−^ by EXTC **(A)**, the Intra-Particle diffusion curves of the uptake reactions **(B)**, Pseudo-First order kinetic modeling **(C)**, and Pseudo-Second order kinetic modeling **(D)**.

A research investigation has been performed to analyze the influence of the contact duration (30–420 min) on the retention properties of EXTC for PO_4_^3-^ and NO_3_^−^. This was completed after establishing the levels of the key variables at specific settings, including pollutants content of 100 mg/L, a pH of 6 for PO_4_^3-^ and 5 for NO_3_^−^, a temperature of 303 K, a volume of 100 mL, and EXTC quantity of 0.2 g/L. The noticeable increase in both the amount of immobilized chemical ions and the discernible rates of retention (Figure 6A) established a significant improvement in the elimination efficacy of EXTC for PO_4_^3-^ and NO_3_^−^ with time. Furthermore, it is essential to acknowledge that the duration of the test has a significant impact on the tracked variations in retention characteristics over time. These variations and associated increments in uptake properties might persist for up to 300 min for PO_4_^3-^ and 200 min for NO_3_^−^ (Figure 6A). However, no appreciable change or enhancement has been observed with regard to the speed of PO_4_^3-^ and NO_3_^−^ ions elimination or the quantity of ions adsorbed following the specified durations. Such experimental findings demonstrate that EXTC could act as a potential adsorbent for PO_4_^3-^ and NO_3_^−^, establishing its equilibrium states within 300 min for PO_4_^3-^ and 200 min for NO_3_^−^. At the equilibrium stage, the EXTC framework had a retention capacity of 115.6 mg/g for PO_4_^3-^ and 88.6 mg/g for NO_3_^−^ (Figure 6A). Throughout the first stages of the experiment, there were significant enhancements and elevated rates of elimination for PO_4_^3-^ and NO_3_^−^, along with greater quantities of retained ions. The enhancements were attributed to the extensive existence of interacted and unbound receptors across the structure of EXTC (El-Sherbeeny et al., 2021b). As the duration of the assessment progressed, there was a distinct drop in the number of available free receptors. The key reason for this tendency could potentially be assigned to the sustained occupation of the adsorbed PO_4_^3-^ and NO_3_^−^ for the present and available binding sites, leading to a decrease in the total number of vacant sites. As a result, there was a significant decrease in the rate of adsorption of PO_4_^3-^ and NO_3_^−^ following a certain period of time. After that, the application of EXTC exhibited little improvement or steady adsorption performances for PO_4_^3-^ and NO_3_^−^, suggesting a condition of stabilization or equilibrium. The equilibrium state of EXTC might be achieved by entirely filling all the active receptors, thereby preventing additional binding of PO_4_^3-^ and NO_3_^−^ across its interface (Allah et al., 2023).

#### 3.2.3 Kinetic studies

##### 3.2.3.1 Intra-particle diffusion behavior

Examining the features of intra-particle diffusion curves may highlight the regulated mechanisms, as well as the retention behaviors of PO_4_^3-^ and NO_3_^−^ using EXTC. The displayed curves exhibit three separate segments with varying slopes (Figure 6B). The analyzed curves demonstrate shifts from the starting point, suggesting the combined existence of multiple adsorption mechanisms together with the diffusion pathways of PO_4_^3-^ and NO_3_^−^ (Abukhadra et al., 2020; El Qada, 2020). The uptake processes generally encompass three major stages: 1) the adsorption of soluble chemical by existing receptors across the exterior interfaces of EXTC (boundary or external retention); 2) the layered adsorption of the chemical ions (inner retention) together in conjunction with the diffusion influence of such ions; and 3) the influence of equilibrium states and saturation conditions (Lin et al., 2021). The early observations obtained from the investigation suggest that the main processes involved in binding PO_4_^3-^ and NO_3_^−^ to EXTC are external retention mechanisms and possess the most effective role throughout the different phases of the retention processes (Figure 6B). During this phase, the efficacy of PO_4_^3-^ and NO_3_^−^adsorption is dependent on the overall number of sites available across the exterior interfaces of EXTC (Chen et al., 2021). The efficiency of the layered adsorption mechanism was promptly demonstrated by extending the period whenever all the external uptake sites had been entirely filled with PO_4_^3-^ and NO_3_^−^ (Figure 6B) (Lin et al., 2021). Furthermore, during this phase, the impact of PO_4_^3-^ and NO_3_^−^ diffusion paths should be considered. Once equilibrium is established, the third segment recognizes the final operating mechanism of PO_4_^3-^ and NO_3_^−^ retention by EXTC and exerts the primary impact. This phase suggests that both PO_4_^3-^ and NO_3_^−^ ions have effectively filled all of the accessible binding sites (Abdel Salam et al., 2022). Molecular and interionic attraction mechanisms facilitate the removal of PO_4_^3-^ and NO_3_^−^ throughout this stage (Allah et al., 2023).

##### 3.2.3.2 Kinetic modeling

The kinetic analysis of the uptake reactions, based on common kinetic models, holds significant importance in illustrating the time-dependent adsorption effects and comprehending the regulated and effective mechanistic processes, such as mass transfer or chemical-based mechanisms (Neolaka et al., 2023). The kinetic properties and behaviors of the elimination processes of PO_4_^3-^ and NO_3_^−^ using EXTC were analyzed using the conventional kinetic theories of pseudo-first-order (P.F.) (Figure 6C) and pseudo-second-order (P.S.) (Figure 6D) mathematical models. The P.F. analysis was applied to evaluate the kinetics of the PO_4_^3-^ and NO_3_^−^ uptake behaviors under stability situations in order to demonstrate the relationship between the speed by which these ions occupy the reactive binding sites and total amounts of their adsorbed ions (Neolaka et al., 2023). It is possible to use the P.S. principles to illustrate the relationship between the characteristics of assessed adsorbents over a specific timeframe. The correlation levels between the experimental behaviors of the retention reactions and kinetic theories were analyzed through the nonlinear fitting parameters associated with the model descriptive equations. The greatest degrees of agreement were identified through analyzing the correlation coefficients (*R* ^2^) alongside Chi-squared (*X* ^*2*^) values (Table 1; Figures 6C, D).

**TABLE 1:** The theoretical parameters of the assessed kinetic models.

Model	Parameters	PO_4_^3-^	NO_3_^−^
Pseudo-First-order	K_1_ (1/min)	0.0026	0.0023
	Qe _(Cal)_ (mg/g)	132.8	113.5
	R^2^	0.89	0.87
	X^2^	6.8	7.1
Pseudo-Second-order	k_2_ (mg/g min)	3.92 E-6	3.75 E-6
	Qe _(Cal)_ (mg/g)	368.8	321.7
	R^2^	0.87	0.84
	X^2^	7.2	7.32

The basic assumptions of the P.F. model (Figure 6C) provide more accurate insights into the binding processes and sequestration characteristics of PO_4_^3-^ and NO_3_^−^ using EXTC, compared to the assessed P.S. assumption, which is based on both *R* ^2^ and *X* ^*2*^ data (Table 1). The estimated equilibrium PO_4_^3-^ and NO_3_^−^ sequestration capacities, using EXTC as a theoretical parameter (132.8 mg/g (PO_4_^3-^) and 113.5 mg/g (NO_3_^−^), were consistent with the experimentally measured quantities. The revealed agreement confirms the earlier stated results, which highlight the greater applicability of the P.F. theory in representing the kinetic features of the retention processes of PO_4_^3-^ and NO_3_^−^ (Table 1). According to the P.F. theory, these two ions were bound to the interfaces of EXTC particles by physical mechanisms such as van der Waals forces and/or electrostatic attraction (Sherlala et al., 2019; Huang et al., 2018). The P.F. model achieves a higher level of consistency, but the examined kinetic simulation factors also demonstrate a significant degree of fitness for the P.S. principles. Recent studies have shown that certain chemical processes, including complexation, ion exchange, and hydrophobic interactions, could potentially enhance the adsorption of PO_4_^3-^ and NO_3_^−^ by EXTC. Physically adsorbed layers could have been developed successively on top of chemically bonded PO_4_^3-^ and NO_3_^−^ layers that have previously been produced (Jasper et al., 2020).

#### 3.2.4 Influence of initial concentration

**FIGURE 7:**
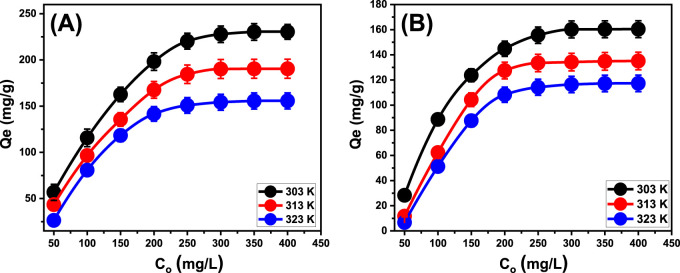
The experimental impact of starting PO_4_^3-^ **(A)** and NO_3_^−^ **(B)** concentrations on their uptake performances by EXTC.

The experiment evaluated the manner in which the starting contents of the soluble chemical affect the effectiveness of their removing using EXTC. It also investigated the equilibrium characteristics within the tested concentrations, which ranged from 50 to 400 mg/L. The factors influencing the removal of PO_4_^3-^ and NO_3_^−^ were maintained at constant levels. The values included an overall volume of 100 mL, duration of 24 h, a pH of 6 for PO_4_^3-^ and 5 for NO_3_^−^, a dosage of 0.2 g/L, and temperatures varying between 303 K and 323 K. An association has been detected between the increased contents of PO_4_^3-^ and NO_3_^−^ ions and the verified increase in their adsorbed quantities by EXTC (Figures 7A, B). The increase in the contents of the tested soluble chemical ions within a certain volume greatly boosted their diffusion and transportation characteristics. This increased the capacity for interaction with a larger number of reactive binding sites, which are frequently distributed throughout the surfaces of EXTC particles. Consequently, increasing their initial concentrations greatly enhanced the efficacy of adsorbing PO_4_^3-^ and NO_3_^−^ using EXTC (Abdel Salam et al., 2022). However, this relationship could only be observed within specific limits for the incorporated PO_4_^3-^ and NO_3_^−^ concentrations (300 mg/L). Subsequently, increasing the initial contents of the two pollutants appears to have no effect on their uptake performances and displays the equilibrium situations of EXTC particulates. Establishing equilibrium states assists in the estimation of the maximum sequestration performance of PO_4_^3-^ and NO_3_^−^. The determined greatest capacities for PO_4_^3-^ were 230.4 mg/g (303 K), 190.5 mg/g (313 K), and 155.7 mg/g (323 K) (Figure 7A). The measured capacities of NO_3_^−^ were 160.4 mg/g at 303 K, 135.1 mg/g at 313 K, and 117.3 mg/g at 323 K (Figure 7B). The decrease in PO_4_^3-^ and NO_3_^−^ removal while applying EXTC at varying temperatures indicates that the uptake processes are exothermic.

#### 3.2.5 Classic isotherm models

**FIGURE 8:**
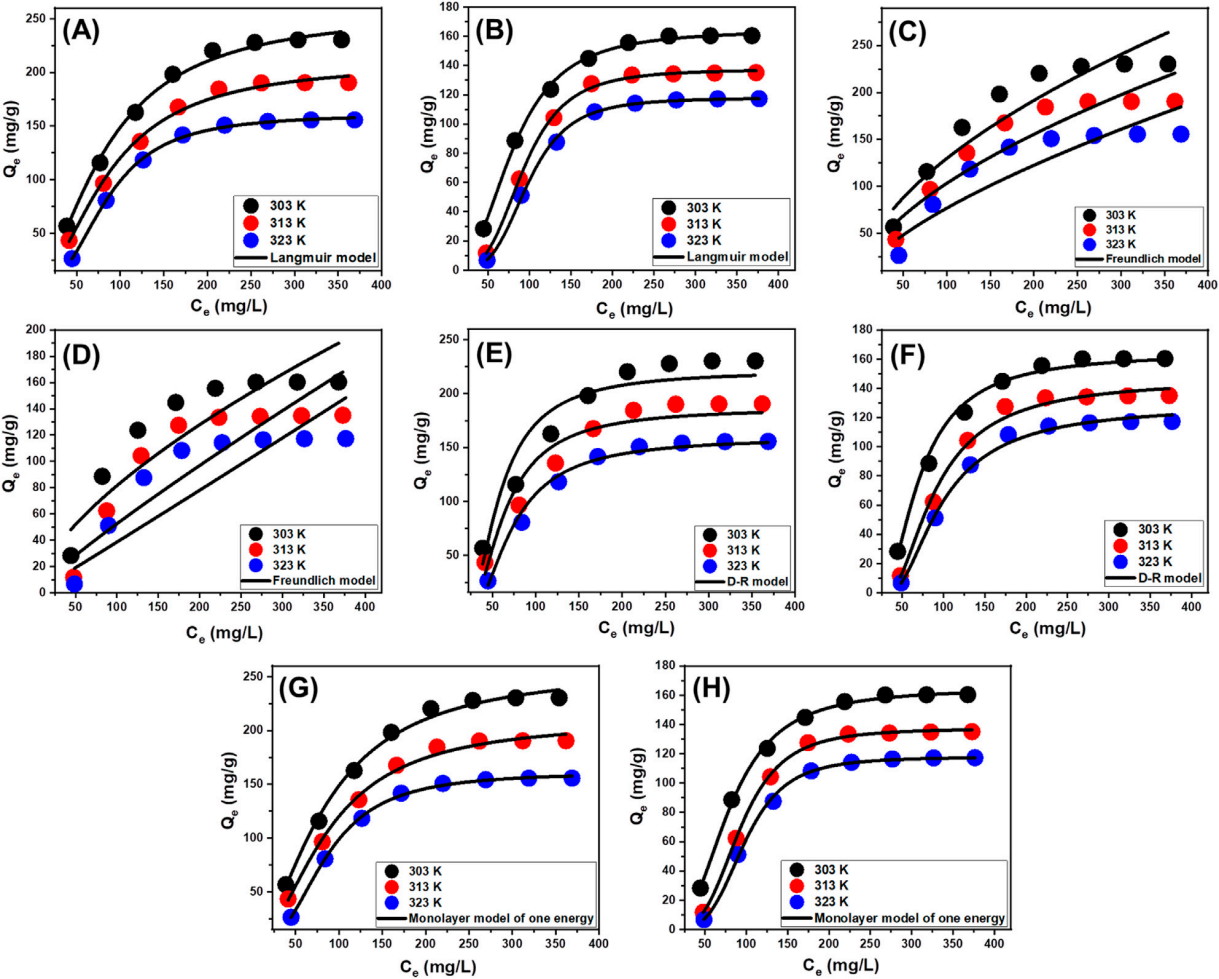
Fitting of the uptake results by EXTC with classic Langmuir model (PO_4_^3-^ **(A)** and NO_3_^−^ **(B)**), Freundlich model (PO_4_^3-^ **(C)** and NO_3_^−^ **(D)**), D-R model (PO_4_^3-^ **(E)** and NO_3_^−^ **(F)**), and advanced monolayer model of one energy site (PO_4_^3-^ **(G)** and NO_3_^−^ **(H)**).

The equilibrium analyses based on classic isotherm principles were performed to assess the distribution of the dissolved chemical ions as contaminants and the interfaces of the applied materials as adsorbents during their stabilization or saturation phase. Classical isotherm models greatly influence the understanding of the mechanistic pathways that regulate uptake activities. The commonly employed isotherm functions offer valuable knowledge in three significant aspects: (a) the selectivity of the adsorbent interface for the tested sorbates; (b) the theoretical quantities of dissolved ions that might potentially interact with the interfaces of the adsorbents; and (c) the maximum possible amount of ions that can be adsorbed. The investigation evaluated the binding reactions of PO_4_^3-^ and NO_3_^−^ by examining their equilibrium characteristics employing the classic Langmuir (Figures 8A, B), Freundlich (Figures 8C, D), and Dubinin-Radushkevich (D-R) (Figures 8E, F) isotherm theories. The alignment between the implied equilibrium assumptions described in the earlier reported models and the measured retention behaviors of PO_4_^3-^ and NO_3_^−^ was assessed using non-linear fitting methods. The investigation included examining the correlation coefficient (*R* ^2^) along with the Chi-squared (*X* ^*2*^) outcomes (Table 2). The analysis of *R* ^2^ and *X* ^*2*^ demonstrates that the EXTC particulates have a greater potential to adsorb PO_4_^3-^ and NO_3_^−^ ions, according to Langmuir’s concepts instead of the Freundlich theory. This suggests that PO_4_^3-^ and NO_3_^−^ exhibit homogenous and uniform adsorption characteristics on the evenly distributed reacting and free sites throughout the EXTC particulates. This leads to the formation of a single layer or monolayers of adsorbed PO_4_^3-^ and NO_3_^−^ (Sherlala et al., 2019; Huang et al., 2018; Jasper et al., 2020). In addition, the investigation showed that EXTC particles exhibited favorable retention properties for PO_4_^3-^ and NO_3_^−^, which was evident by the RL values being lower than 1 (Table 2) (Abdel Salam et al., 2022; Allah et al., 2023). The theoretical analysis revealed that the maximum adsorption capacities (Q_max_) for PO_4_^3-^ were 257.9 mg/g at 303 K, 209.2 mg/g at 313 K, and 160.9 mg/g at 323 K. The theoretical values for NO_3_^−^ are 164.2 mg/g at 303 K, 137.3 mg/g at 313 K, and 117.8 mg/g at 323 K.

**TABLE 2:** The theoretical parameters of the assessed classic and advanced isotherm models.

Model	Parameter	PO_4_^3-^			NO_3_^−^		
		303 K	313 K	323 K	303 K	313 K	323 K
Langmuir	Q_max_ (mg/g)	257.9	209.2	160.9	164.2	137.3	117.8
	b(L/mg)	5.36 E-4	2.17 E-4	9.54 E-6	8.48 E-6	8.12 E-8	1.38 E-8
	R^2^	0.99	0.99	0.99	0.99	0.99	0.99
	X^2^	0.19	0.16	0.02	0.04	0.025	0.42
Freundlich	1/n	0.56	0.59	0.67	0.65	0.88	0.91
	k_F_ (mg/g)	9.8	6.8	3.5	4.1	0.89	0.36
	R^2^	0.90	0.89	0.85	0.84	0.81	0.81
	X^2^	3.7	3.6	5.25	6.5	8.2	8.8
D-R model	β (mol^2^/KJ^2^)	0.0072	0.0078	0.0084	0.008	0.009	0.011
	Q_m_ (mg/g)	231.3	196.8	159.2	164.2	146.0	128.1
	R^2^	0.94	0.95	0.99	0.99	0.99	0.99
	X^2^	2.8	2.4	0.40	0.21	0.21	0.12
	E (KJ/mol)	8.3	8.0	7.71	7.9	7.4	6.7
Monolayer model of one energy site	n	1.7	1.9	2.6	2.6	3.6	3.9
	Nm (mg/g)	151.5	110.3	61.6	61.5	38.0	29.8
	Q_sat_ (mg/g)	257.9	209.2	160.9	164.2	137.3	117.8
	C1/2 (mg/L)	83.4	85.4	89.6	79.2	92.2	97.5
	ΔE (kJ/mol)	−3.28	−3.45	−3.69	−3.16	−3.66	−3.92

The equilibrium characteristics of the D-R theory provide considerable explanation for the energetic fluctuations displayed by EXTC particles during the elimination of PO_4_^3-^ and NO_3_^−^, regardless of the particle’s homogeneity level (Dawodu et al., 2012). The investigation of the D-R modeling outcomes gives useful insights into the estimated value of Gaussian energy levels (E) and their role in identifying the regulated mechanistic activities, irrespective of whether these processes are chemical or physical. The energetic levels corresponding to the retention processes can possibly be classified into three separate categories: those below 8 kJ/mol, those between 8 and 16 kJ/mol, and those beyond 16 kJ/mol. At such energies, the primary mechanisms are predominantly physical, faint chemical, or combinations of physical and chemical pathways, and finally strong chemical mechanisms (El-Sherbeeny et al., 2021b). The measured E readings for the uptake of PO_4_^3-^ and NO_3_^−^ utilizing EXTC have been determined to be inside the energy ranges of physical processes and slightly match the suggested limits of complex physical and weak chemical mechanisms (Table 2).

#### 3.2.6 Advanced isotherm models

Applying statistical physics principles to predict the equilibrium functions of adsorption activities facilitates an in-depth assessment of the distinctive characteristics of such processes. The current investigation uses mathematical models derived from statistical physics to track the interactions between water-soluble contaminants and exterior-reacting chemical structures. The existing active chemical groups act as binding sites on the surfaces of the adsorption products. The mathematical equations used in this study provide accurate estimations for several parameters that effectively represent the key activities, including both energetic and steric factors. The mathematical models involve many steric factors, such as Nm, which quantifies the total number of filled adsorption sites throughout the structure of EXTC. In addition, the calculations demonstrate the number of chemical ions (n) that only one receptor can absorb, along with the highest possible level of PO_4_^3-^ and NO_3_^−^ sequestration utilizing EXTC whenever they achieve complete saturation (Q_sat_). The energetic aspects comprise the internal energy (E_int_), entropy (Sa), adsorption energies (E), and enthalpy (G). Non-linear regression analysis assessed the assumptions of the developed models. Multivariable nonlinear regression methodologies, together with the Levenberg-Marquardt iterative method, successfully accomplished the previous inquiry. The established fitting levels were then implemented to assess and characterize the adsorption properties of EXTC. The most comparable model—the monolayer model with a single active site—was used to represent the PO_4_^3-^ and NO_3_^−^ adsorption reactions (Figures 8G, H). Table 2 displays the computed parameters from the assessed model.

##### 3.2.6.1 Steric properties

###### 3.2.6.1.1 Number of adsorbed metal ions per site (n)

**FIGURE 9:**
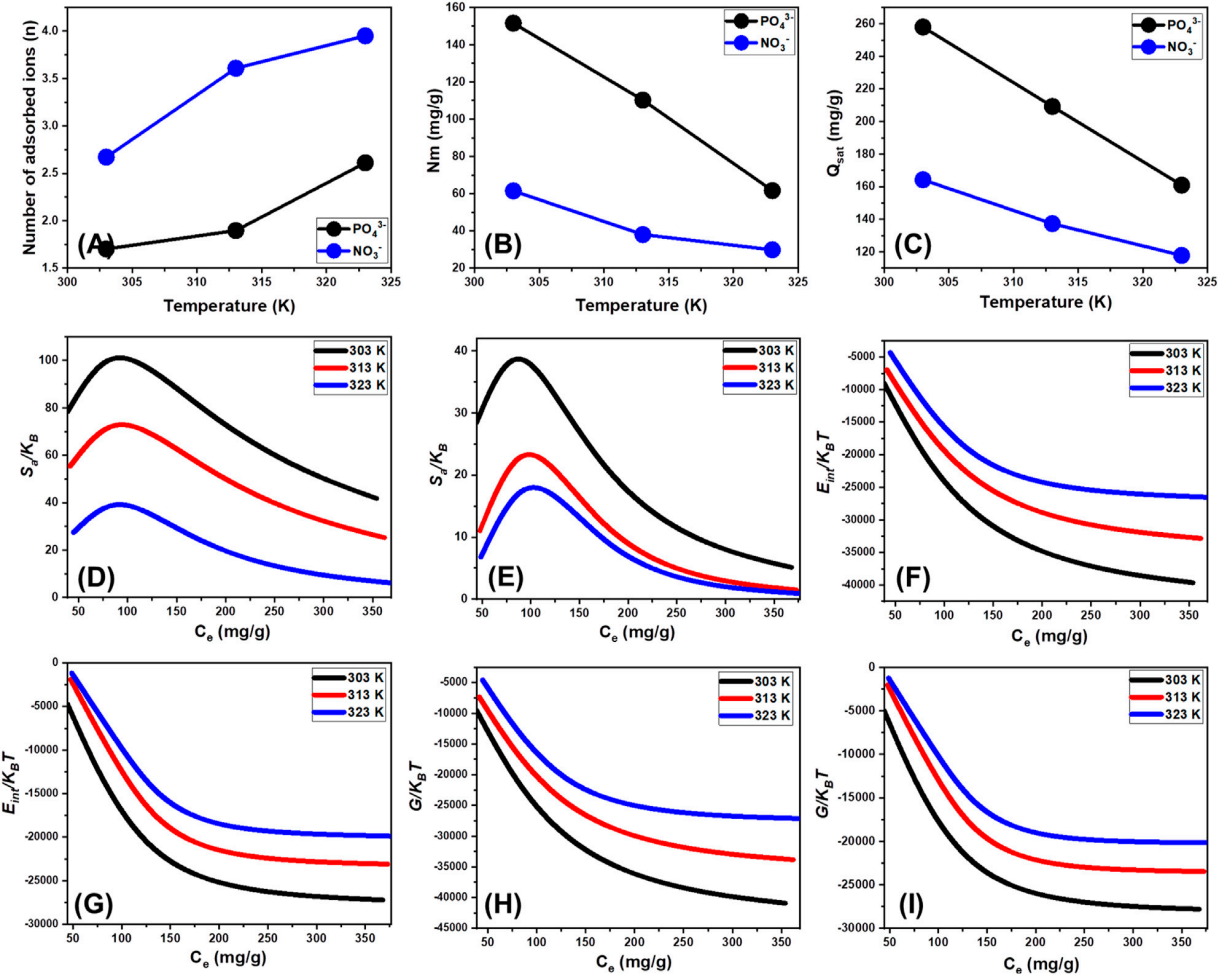
Changes in the steric parameters during the uptake of PO_4_^3-^ and NO_3_^−^ (number of adsorbed ions **(A)**, active sites density **(B)**, and saturation uptake capacity **(C)**) and thermodynamic functions (Entropy **(D)** (PO_4_^3-^) and **(E)** (NO_3_^−^)), internal energy **(F)** (PO_4_^3-^) and **(G)** (NO_3_^−^)), and enthalpy **(H)** (PO_4_^3-^) and**(I)** (NO_3_^−^)) in terms of the concentration and temperature.

The mathematical insights derived from the n parameter offer a valuable understanding of the distribution pattern of the bound PO_4_^3-^ and NO_3_^−^ ions throughout the external interfaces of the EXTC particles. This encompasses either vertical or horizontal configurations. Furthermore, the consequences of these results are very valuable in comprehending the mechanisms that govern adsorption behaviors, which include multi-docking and multi-interactions. Among the reactions that are greatly affected by multi-anchorage or multi-docking processes is the acquisition of a single PO_4_^3-^ or NO_3_^−^ ion within several uptake receptors. Such adsorption reactions have n levels below 1 and correlate with the horizontal arrangement of these adsorbed ions. In contrast, processes with levels greater than 1 suggest the retention of PO_4_^3-^ and NO_3_^−^ through non-parallel configurations and vertical orientations. Adsorption mechanisms operated during these types of reactions are mostly facilitated by multi-ionic interaction processes, in which a single receptor sequesters multiple metal ions (Abdel Salam et al., 2022; Mobarak et al., 2021). The estimated values of n, which describe the overall amount of metal ions bound onto an individual adsorption site through the outer surface of EXTC, vary between 1.7 and 2.6 for PO_4_^3-^ and between 2.6 and 3.9 for NO_3_^−^ (Figure 9A; Table 2). Values greater than 1 are indicative of the sequestration of both PO_4_^3-^ and NO_3_^−^ via multi-ionic interactions. Each uptake site inside the EXTC particles exhibited the capacity to adsorb 3 ions of PO_4_^3-^ and up to 4 ions of NO_3_^−^ as individual ions. This suggests the higher aggregation behaviors of NO_3_^−^ ions across the interface of EXTC as compared to PO_4_^3-^ or the higher interaction activities of the phosphate ions with the higher numbers of silicate functional groups. These adsorbed ions either PO_4_^3-^ or NO_3_^−^ exhibited vertical configurations with non-parallel properties. As the temperature increases from 303 K to 323 K, the computed values of the n parameter either during the uptake of PO_4_^3-^ and NO_3_^−^ increase at considerable rates (Figure 9A; Table 2). The expected elevation in the aggregating properties of the PO_4_^3-^ and NO_3_^−^ ions during their interactions with the external layer of EXTC at high temperatures might potentially explain the reported findings (Ali et al., 2021).

###### 3.2.6.1.2 Density of the active sites (Nm)

The average density of the active sites could theoretically be used to precisely quantify the quantities of existing binding sites that have been occupied with PO_4_^3-^ and NO_3_^−^ throughout the surfaces of EXTC particulates. The estimated Nm values of EXTC at different temperature conditions for PO_4_^3-^ were as follows: 151.6 mg/g at 303 K, 110.3 mg/g at 313 K, and 61.6 mg/g at 323 K (Figure 9B; Table 2). The obtained values for NO_3_^−^ were 61.5 mg/g at 303 K, 38 mg/g at 313 K, and 29.8 mg/g at 323 K. This supports the earlier suggesting about the higher interaction activities of the phosphate ions with the structure of EXTC as comparted to nitrate as they have tendency to form strong inner sphere complexes. Temperature effects are evident in the quantities of Nm across the exterior of EXTC following the sequestration processes for such ions, which show reversible changes with the operation temperature (Figure 9B; Table 2). The noticed behaviors correlate with the previously established characteristics of n, since the increased ability of PO_4_^3-^ and NO_3_^−^ to aggregate results in a decrease in the overall number of occupied sites. This characteristic can be reinforced by the expected effect of temperature on the activation degrees of the distributed retention sites (Yang et al., 2022; Jasper et al., 2020). The analysis demonstrates the adverse impact of rising temperatures on the occupancy of sites. This impact may occur either by deactivating particular functioning sites over time or by reducing the time required for these sites to successfully absorb ions. Previous investigations have shown similar characteristics, which may be attributed to the suggested desorption and release of the chemical ions that had previously been bound to the surface of EXTC. The desorption response was a result of the decrease in saturation constraints of heated solutions.

###### 3.2.6.1.3 Adsorption capacity at the saturation state of (Q_sat_)

The saturation adsorption characteristics of EXTC (Q_sat_) provide the most effective adsorption of PO_4_^3-^ and NO_3_^−^, along with the best degree of tolerance. Two important parameters influence the computation of Q_sat_ values: the specified density of filled sites (Nm) and the aggregate quantity of metal ions trapped into each distinct site (n). For PO_4_^3-^, the saturation retention efficiency of EXTC has been estimated to be 257.9 mg/g at 303 K, 209.2 mg/g at 313 K, and 160.9 mg/g at 323 K. For NO_3_^−^, the values were found to be 164.2 mg/g at a temperature of 303 K, 137.3 mg/g at 313 K, and 117.8 mg/g at 323 K (Figure 9C; Table 2). While charge density plays a major role in the preferential adsorption of PO_4_^3-^ over NO_3_^−^, hydration energy, coordination chemistry, and steric effects also significantly contribute to this selectivity. The combination of inner-sphere complexation, ion-exchange interactions, and nanoconfinement effects leads to stronger phosphate retention on EXTC, while nitrate primarily interacts via weaker electrostatic and hydrogen bonding mechanisms. PO_4_^3-^ carries a higher negative charge compared to NO_3_^−^, which inherently increases its electrostatic attraction to positively charged adsorption sites on EXTC. Additionally, the hydration radius of PO_4_^3-^ is smaller than that of NO_3_^−^, which allows phosphate to access adsorption sites more efficiently, particularly within the nanoconfined regions of exfoliated silicate sheets. Steric factors also influence the adsorption selectivity of EXTC. PO_4_^3-^, with its tetrahedral geometry, fits more effectively into the adsorption sites of EXTC, particularly in nanoconfined regions. NO_3_^−^, being planar, experiences weaker binding forces and is more prone to desorption under competitive conditions.

The negative temperature-related findings suggest that the EXTC-based PO_4_^3-^ and NO_3_^−^ sequestration mechanisms are exothermic. The findings indicate that higher sequestration temperatures lead to a higher prevalence of thermal collisions, thereby decreasing the binding effectiveness of PO_4_^3-^ and NO_3_^−^ (Mobarak et al., 2021). Furthermore, Q_sat_’s temperature-dependent detectable characteristics bear resemblance to the behavior elucidated by Nm instead of n. The obtained results suggest that the overall number of interacting sites, rather than the specific binding capacity of each individual receptor, primarily influences the effectiveness of PO_4_^3-^ and NO_3_^−^ adsorption.

##### 3.2.6.2 Energetic properties

###### 3.2.6.2.1 Adsorption energy

The evaluation of adsorption energy fluctuations (ΔE) during the uptake of phosphate (PO_4_^3-^) and nitrate (NO_3_^−^) provides essential insights into the underlying mechanisms governing ion sequestration, distinguishing between physical and chemical adsorption interactions. Typically, adsorption energy values below 40 kJ/mol indicate physical adsorption processes, whereas values exceeding 80 kJ/mol suggest a dominant chemical mechanism. Physical adsorption mechanisms can be further classified based on their characteristic energy levels, which include hydrogen bonding (<30 kJ/mol), electrostatic attractions (2–50 kJ/mol), dipole interactions (2–29 kJ/mol), van der Waals forces (4–10 kJ/mol), and hydrophobic interactions (∼5 kJ/mol). The computed adsorption energy values, derived using Equation 5, incorporate key thermodynamic parameters such as the solubility (S) of the adsorbate, the universal gas constant (R = 0.008314 kJ/mol·K), the ion concentration at half-saturation (C), and the absolute temperature (T) (Dhaouadi et al., 2020).

$$∆E=RT\;\ln\left(\frac{S}{C}\right)$$ (5)

The obtained ΔE values for EXTC adsorption ranged from −3.28 to −3.69 kJ/mol for PO_4_^3-^ and from −3.16 to −3.92 kJ/mol for NO_3_^−^ (Table 2). These energy values confirm that the adsorption of both anions is primarily governed by physical interactions rather than chemical bonding. The dominant mechanisms facilitating ion uptake include dipole bonding (2–29 kJ/mol), electrostatic attractions (2–50 kJ/mol), van der Waals forces (4–10 kJ/mol), and hydrogen bonding (<30 kJ/mol). These findings highlight the efficiency of EXTC as a selective adsorbent for phosphate and nitrate removal through predominantly physical interactions. The low-energy adsorption process ensures reversible ion binding, enhancing the material’s reusability and practical applicability in water treatment systems. Furthermore, the reliance on weak interactions reduces the likelihood of structural degradation, extending the material’s operational lifespan and minimizing the need for chemical regeneration treatments. The adsorption energy values correlate closely with the thermodynamic functions, further supporting the classification of EXTC’s adsorption mechanism as primarily physical. The negative values of ΔE indicate an exothermic and spontaneous adsorption process, confirming that no significant chemical bond formation occurs between the adsorbent and the target anions.

Based on the FT-IR findings in conjunction with the significances of the adsorption energy, the electrostatic attractions of PO_4_^3-^ and NO_3_^−^ ions by EXTC occurs between these negatively charged anions and positively charged Mg^2+^ and silanol (-SiOH) groups. The hydrogen bonding might be occurred as a result of the interactions with hydroxyl (-OH) functional groups. Also, these ions might be formed complexs with surface-exposed silicate and magnesium sites. Furthermore, the ion exchange interaction might be facilitated by the structural reorganization of Mg–OH and Si–O–Mg groups.

###### 3.2.6.2.2 Thermodynamic functions

####### 3.2.6.2.2.1 Entropy

The entropy (Sa) of PO_4_^3-^ and NO_3_^−^ through the adsorption reactions using EXTC demonstrates the unique characteristics of surface order and disorder that define their external interfaces at varying metal ion concentrations and temperatures. The features of Sa were examined based on the outcomes of Equation 6 (Dhaouadi et al., 2020), which comprised the previously calculated values for Nm and n, as well as the predicted concentrations of PO_4_^3-^ and NO_3_^−^ at the half-saturation states (C1/2) and Boltzmann constant (*K*_*B*_ = 1.380 × 10^−23^ m^2^.*Kg*.*s* ^-2^.*K* ^−1^).

$$\frac{S_{a}}{K_{B}}=Nm\left\{\ln\left(1+{\left(\frac{C}{C_{\frac{1}{2}}}\right)}^{n}\right)-n{\left(\frac{C}{C_{\frac{1}{2}}}\right)}^{n}\;\frac{\ln\left(\frac{C}{C_{\frac{1}{2}}}\right)}{1+{\left(\frac{C}{C_{\frac{1}{2}}}\right)}^{n\;}}\;\right\}$$ (6)

A detailed examination of the generated graphs reveals a notable decline in entropy (Sa) upon the adsorption of phosphate (PO_4_^3-^) and nitrate (NO_3_^−^) ions onto EXTC, particularly at elevated initial concentrations of these anions (Figures 9D, E). This trend signifies a substantial reduction in the degree of randomness at the solid-liquid interface as ion adsorption progresses. The results indicate that as the concentrations of PO_4_^3-^ and NO_3_^−^ increase, the adsorption interface of EXTC undergoes a marked decrease in disorder. This suggests a significant enhancement in the binding efficiency of the active functional groups within EXTC, even when the anion concentrations remain relatively low (Jasper et al., 2020; Dawodu et al., 2012). For PO_4_^3-^ adsorption, the highest recorded entropy values corresponded to equilibrium concentrations of 76.8 mg/L at 303 K, 80.7 mg/L at 313 K, and 83.8 mg/L at 323 K (Figure 9D). Similarly, NO_3_^−^ exhibited peak entropy values at equilibrium concentrations of 82.3 mg/L at 303 K, 87.5 mg/L at 313 K, and 89.7 mg/L at 323 K (Figure 9E). These concentrations closely align with the half-saturation adsorption stages of EXTC, reinforcing the idea that as the available active sites become increasingly occupied, additional ion binding becomes more restricted. This restriction leads to a more structured adsorption layer, thereby decreasing the configurational entropy of the system. The pronounced reductions in calculated entropy values further indicate a significant decline in the number of accessible adsorption sites, coupled with a substantial decrease in the mobility and diffusional freedom of the adsorbed ions (Sellaoui et al., 2016). This entropy reduction can be attributed to multiple factors, including the formation of stronger ion-surface interactions at higher ion loadings, the compact structuring of the adsorbed layers, the enhancement in multi-interactions chances, and the dehydration effects of ions upon adsorption. Additionally, at elevated temperatures, enhanced ion binding stability and reduced solvation effects contribute to a more ordered adsorption system, further explaining the inverse relationship between temperature and entropy. These findings highlight the structural and thermodynamic intricacies of EXTC as an advanced adsorbent, providing key insights into its applicability for selective phosphate and nitrate removal in environmental remediation.

####### 3.2.6.2.2.2 Internal energy and free enthalpy

Internal energy (E_int_) and free enthalpy properties that correlated with the binding characteristics of PO_4_^3-^ and NO_3_^−^ into EXTC were analyzed with respect to the changes in the metal concentrations and the operation temperature. Equations 7, 8 (Dhaouadi et al., 2021) were used to complete the analysis, employing the translation partition (Zv) and the specified values of Nm, n, and C1/2.

$$\frac{E_{\mathrm{int}}}{K_{B}T\;}=n\;N_{m}\left[\left(\;\frac{{\left(\frac{C}{C_{1/2}}\right)}^{n}\;\ln\left(\frac{C}{Z_{v}}\right)}{1+{\left(\frac{C}{C_{1/2}}\right)}^{n}}\right)-\left(\;\frac{n\ln\left(\frac{C}{C_{1/2}}\right)\;{\left(\frac{C}{C_{1/2}}\right)}^{n}}{1+{\left(\frac{C}{C_{1/2}}\right)}^{n}}\right)\right]$$ (7)

$$\frac{G}{K_{B}T\;}=n\;N_{m}\frac{\ln\left(\frac{C}{Z_{v}}\right)}{1+{\left(\frac{C_{1/2}}{C}\right)}^{n}}$$ (8)

The investigation of the changes in E_int_ with respect to the elimination characteristics of PO_4_^3-^ and NO_3_^−^ employing EXTC reveals values with negative signs. The findings demonstrate a significant decline in E_int_ with increasing temperature from 303 K to 323 K (Figures 9F, G). The results obtained here indicate that the PO_4_^3-^ and NO_3_^−^ sequestration processes by EXTC are spontaneous and exothermic. The observed decrease in internal energy at higher adsorption capacities further confirms the exothermic nature of the process, where adsorbed ions stabilize at the surface without inducing significant structural modifications in EXTC. The enthalpy evaluations and behaviors reflect the same criteria and features as the internal energy. The G results show negatively signed values and a reversible correlation with the specific binding temperature (Figures 9H, I). These findings indicate a reduction in the feasibility criteria and confirm the exothermic and spontaneous behavior of the PO_4_^3-^ and NO_3_^−^ ions retention processes using EXTC. The adsorption enthalpy remains within a range characteristic of weak physical interactions, indicating that the process is driven by van der Waals forces and hydrogen bonding rather than stronger chemisorption mechanisms which are in agreement with the estimated adsorption energies.

#### 3.2.7 Recyclability

**FIGURE 10:**
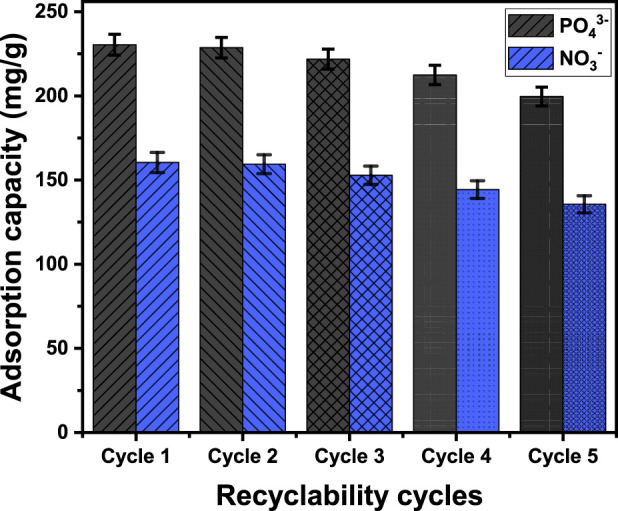
The recyclability properties of synthetic EXTC during the removal of PO_4_^3-^ and NO_3_^−^.

The evaluation of EXTC’s value for commercial and practical applications significantly depends on its potential for recycling as an adsorbent. The regenerative process used to purify the previously used EXTC particulates consisted of a thorough washing stage employing an orbital shaker at room temperature for 30 min. Subsequently, the regenerated particulates were sterilized by the use of distilled water before being dried for a duration of 12 h at 50°C. The two different metals had been subjected to repeated adsorption studies under controlled experimental conditions. The specified conditions were: a total volume of 100 mL, a 24-h interval, a pH of 6 for PO_4_^3-^ and 5 for NO_3_^−^, a concentration of 400 mg/L, a dosage of 0.2 g/L, and a temperature of 303 K. The findings emphasize the remarkable recyclability value of EXTC and its significant capacity to be repeatedly employed as an adsorbent for PO_4_^3-^ and NO_3_^−^ under the evaluated levels of concentration (Figure 10). The effectiveness of PO_4_^3-^ elimination in Run 1, Run 2, Run 3, Run 4, and Run 5 was 230.4 mg/g, 228.6 mg/g, 221.8 mg/g, 212.4 mg/g, and 199.6 mg/g, respectively (Figure 10). For NO_3_^−^, the values determined were 160.4 mg/g, 159.4 mg/g, 152.8 mg/g, 144.3 mg/g, and 135.6 mg/g for Runs 1, 2, 3, 4, and 5 (Figure 10). The adsorption affinity of EXTC for the two metals declined as the number of recycle and reuse cycles increased. This trend demonstrates the continuous development of chemical complexes, in which the essential functional chemical groups of EXTC interact with the adsorbed ions. The presence of these complexes has a detrimental impact on the total number of accessible, unbound, and active sites following each reuse experiment.

#### 3.2.8 Comparison study

The exfoliated nanomagnesium silicate sheets (EXTC) demonstrated exceptional adsorption efficiency compared to raw talc and various conventional adsorbents, making it a highly promising material for the removal of phosphate (PO_4_^3-^) and nitrate (NO_3_^−^) from aqueous solutions. The maximum adsorption capacities (Q_max_) of EXTC for PO_4_^3-^ (257.9 mg/g) and NO_3_^−^ (164.2 mg/g) significantly surpass those of unmodified talc (61.2 mg/g for PO_4_^3-^ and 36.4 mg/g for NO_3_^−^), as well as other commonly employed adsorbents, including zeolites, biochars, metal-organic frameworks (MOFs), layered double hydroxides (LDHs), and graphene-based materials. These findings highlight the effectiveness of EXTC in addressing environmental challenges associated with anionic contamination. The enhanced adsorption capacity of EXTC can be attributed to the structural and physicochemical modifications induced by the exfoliation process. Compared to conventional adsorbents, EXTC exhibits superior ion-exchange capabilities and electrostatic interactions, which facilitate the effective sequestration of PO_4_^3-^ and NO_3_^−^. The uniform dispersion of adsorption sites ensures a high affinity for these anions, further supporting its application in large-scale wastewater treatment.

Another major advantage of EXTC is its lower dosage requirement and faster adsorption kinetics compared to traditional adsorbents. Due to its higher surface reactivity, a smaller amount of EXTC can achieve pollutant removal levels equivalent to or higher than other adsorbents, leading to reduced material consumption and shorter treatment durations. This efficiency not only enhances the economic feasibility of EXTC but also minimizes operational costs, making it an attractive alternative to synthetic adsorbents such as MOFs and modified LDHs. From an environmental and economic standpoint, EXTC offers a cost-effective and sustainable solution for anionic pollutant removal. Unlike many synthetic adsorbents that rely on chemical-intensive production methods, EXTC is derived from natural talc, an abundant and low-cost mineral.

## 4 Conclusion

Magnesium silicate separated nano-sheets was synthesized effectively by exfoliation and delamination of natural talc mineral. The exfoliated product (EXTC) displayed significant surface area (102 m^2^/g), mesoporous structure (29.5 nm), and enhanced adsorption efficiency for PO_4_^3-^ (257.9 mg/g) and NO_3_^−^ (164.2 mg/g) as water pollutants. These adsorption capacities recommending its application in purification of industrial and agricultural waste water as low cost and effective adsorbent. The structure exhibit significant reusability and its spent product can be applied as natural fertilizer which can be assessed by further optimization studies. These adsorption behaviors follow the Pseudo-First order kinetics and classic Langmuir isotherm. The steric investigation declared the higher existed of interactive sites during the uptake of PO_4_^3-^ (151.5 mg/g) than NO_3_^−^ (61.5 mg/g) explaining the better uptake efficiency and aggregation tendency (n = 4 for NO_3_^−^ and 3 for PO_4_^3-^). Such steric findings in addition to energetic factors (E < 8.5 kJ/mol and ΔE < 4 kJ/mol) and thermodynamic functions reveal the sequestration of the two ions into EXTC through spontaneous, physical, and exothermic processes.

## Data Availability

The original contributions presented in the study are included in the article/Supplementary Material, further inquiries can be directed to the corresponding author.
