# Supplementary material for: Facile exfoliation of natural talc into separated mesoporous magnesium silicate nano-sheets for effective sequestration of phosphate and nitrate ions: characterization and advanced modeling
Source: Front Chem. 2025 Apr 15;13:1571723. doi: 10.3389/fchem.2025.1571723 (PMC12037480; doi:10.3389/fchem.2025.1571723)
Supplement: Supplementary file 1 [file Table1.docx]

Table.S1. Nonlinear equations of kinetic, classic isotherm, and advanced isotherm models

| Kinetic models | | |
| --- | --- | --- |
| Model | Equation | Parameters |
| Pseudo-first-order | $Q_{t}=Q_{e} (1-e^{{-k}_{1}.t})$ | Q_t_ (mg/g) is the adsorbed ions at time (t), and K_1_ is the rate constant of the first-order adsorption (1/min) |
| Pseudo-second-order | $Q_{t}=\frac{Q_{e}^{2}k_{2}t}{1+Q_{e}k_{2}t}$ | Qe is the quantity of adsorbed ions after equilibration (mg/g), and K_2_ is the model rate constant (g/mg min). |
| Classic Isotherm models | | |
| Model | Equation | Parameters |
| Langmuir | $Q_{e}=\frac{Q_{max} bC_{e}}{(1+bC_{e})}$ | *C_e_* is the rest ions concentrations (mg/L), *Q_max_* is the theoritical maximum adsorption capacity (mg/g), and *b* is the Langmuir constant (L/mg) |
| Freundlich | $Q_{e}=K_{f}C_{e}^{1/n}$ | K_F_ (mg/g) is the constant of Freundlich model related to the adsorption capacity and n is the constant of Freundlich model related to the adsorption intensities |
| Dubinin–Radushkevich | $Q_{e}=Q_{m}e^{-\betaɛ^{2}}$ | β (mol^2^/KJ^2^) is the D-R constant, ɛ (KJ^2^/mol^2^) is the polanyil potential, and Q_m_ is the adsorption capacity (mg/g) |
| Advanced isotherm models | | |
| Model | Equation | Parameters |
| Monolayer model with one energy site (Model 1) | $Q=nN_{o} =\frac{nN_{M}}{1+{(\frac{C1/2}{C})}^{n}}=\frac{Q_{o}}{1+{(\frac{C1/2}{C})}^{n}}$ | Q is the adsorbed quantities in mg/g  n is the number of adsorbed ion per site  Nm is the density of the effective receptor sites (mg/g)  Q_o_ is the adsorption capacity at the saturation state in mg/g  C1/2 is the concentration of the ions at half saturation stage in mg/L  C1 and C2 are the concentrations of the ions at the half saturation stage for the first active sites and the second active sites, respectively  n1 and n2 are the adsorbed ions per site for the first active sites and the second active sites, respectively |
| Monolayer model with two energy sites (Model 2) | $Q=\frac{n_{1}N_{1M}}{1+{(\frac{C_{1}}{C})}^{n_{1}}}+\frac{n_{2}N_{2M}}{1+{(\frac{C_{2}}{C})}^{n_{2}}}$ |  |
| Double layer model with one energy site (Model 3) | $Q=Q_{o}\frac{({\frac{C}{C1/2})}^{n}+2({\frac{C}{C1/2})}^{2n}}{1+({\frac{C}{C1/2})}^{n}+({\frac{C}{C1/2})}^{2n}}$ |  |
| Double layer model with two energy sites (Model 3) | $Q=Q_{o}\frac{({\frac{C}{C1})}^{n}+2({\frac{C}{C2})}^{2n}}{1+({\frac{C}{C1})}^{n}+({\frac{C}{C2})}^{2n}}$ |  |

**Table.S2.** Comparison study between the developed EXTC as adsorbent and other adsorbents in literature

| PO_4_^3-^ | | | NO_3_^-^ | | |
| --- | --- | --- | --- | --- | --- |
| Adsorbents | **q_max_ (mg/g)** | **References** | **Adsorbents** | **q_max_ (mg/g)** | **References** |
| Zeolite A | 52.91 | Hamdi and Srasra, (2012) | **Chitosan /ZeoliteY/ ZrO_2_** | 23.58 | Teimouri et al., (2016) |
| MCM-41/Rice husk | 21 | Seliem et al., (2016) | **Al-modified biochar** | 89.5 | Yin et al., (2018) |
| LTA MOFs | 62.8 | Kumar et al., (2021) | **LTA MOFs** | 50.01 | Kumar et al., (2021) |
| Calcined Mg-Al-LDHs | 40.78 | Das et al., (2006) | **PEI–HCl/ cocoa shell** | 86.95 | Fotsing et al., (2020) |
| ZrO_2_ nanoparticles | 99 | Su et al., (2013) | **MK-chitosan** | 74.89 | Karthikeyan and Meenakshi., (2021) |
| Lanthanum hydroxides | 107.5 | Xie et al., (2014) | **Alginate@ZnFe-LDHs** | 74.129 | Karthikeyan and Meenakshi., (2021) |
| Mg(OH)_2_/ZrO_2_ | 87.2 | Lin et al., (2019) | **HDTMA modified zeolite** | 12.35 | Onyango et al., (2010) |
| Titanium modified zeolite | 37.60 | Alshameri et al., (2014) | **Zr@CSBent composite** | 23.9 | Kumar et al., (2020) |
| Al-modified biochar | 57.49 | Yin et al., (2018) | **MXenes** | 70.4 | Karthikeyan et al., (2020) |
| Hydrous zirconium oxide | 51.8 | Lin et al., (2017) | **Biochar /Modified Zeolite** | 24.45 | Wang et al., (2021) |
| Kaolintic clay | 38.46 | Hamdi and Srasra, (2012) | **Triethylamine/Giant reed** | 118.9 | Ren et al., (2016) |
| La_100_SBA-15 | 45.6 | Yang et al., (2011) | **Aminated alkaline lignin** | 111.6 | Orlando et al., (2002) |
| Mg/Al modified biochar | 56.12 | Deng et al., (2021) | **Magnetic cationic hydrogel** | 95.88 | Li et al., (2020) |
| Biochar | 133 | Yao et al., (2011) | **Graphene** | 89.9 | Ganesan et al., (2013) |
| Zirconia/graphite oxide | 149.3 | Zong et al., (2013) | **Fe/Pd bimetal-loaded zeolite** | 99.5 | He et al., (2020) |
| Titania/GO | 33.11 | Sakulpaisan et al., (2016) | **Amine cross-linked tea wastes** | 98.72 | Qiao et al., (2019) |
| La doping magnetic graphene | 116.28 | Nodeh et al., (2017) | **Biochar-supported polyaniline** | 72 | Herath et al., (2021) |
| EXTC | **257.9** | **This study** | **EXTC** | **164.2** | **This study** |
